# Curcumin attenuates inflammation of Macrophage-derived foam cells treated with Poly-L-lactic acid degradation via PPARγ signaling pathway

**DOI:** 10.1007/s10856-022-06654-7

**Published:** 2022-03-18

**Authors:** Dongping Chen, Yangbo Xi, Suzhen Zhang, Linsheng Weng, Zhihui Dong, Can Chen, Tim Wu, Jianmin Xiao

**Affiliations:** 1 Central Laboratory, The Dongguan Affiliated Hospital of Jinan University, Binhaiwan Central Hospital of Dongguan, Dongguan, China; 2 Department of Cardiology, The Dongguan Affiliated Hospital of Jinan University, Binhaiwan Central Hospital of Dongguan, Dongguan, China; 3 Department of The First Clinical Medical College, Jinan University, Guangzhou, China; 4 Intensive Care Unit of Foshan Women and Children Hospital, Foshan, China; 5 Department of pathology, The Dongguan Affiliated Hospital of Jinan University, Binhaiwan Central Hospital of Dongguan, Dongguan, China; 6 Dongguan TT Medical,Inc, Dongguan, China; 7 VasoTech, Inc., Lowell, MA USA

## Abstract

Poly-L-lactic acid (PLLA) is considered to be a promising candidate material for biodegradable vascular scaffolds (BVS) in percutaneous coronary intervention (PCI). But, PLLA-BVS also faces the challenge of thrombosis (ST) and in-stent restenosis (ISR) caused by in-stent neo-atherosclerosis (ISNA) associated with inflammatory reactions in macrophage-derived foam cells. Our previous studies have confirmed that curcumin alleviates PLLA-induced injury and inflammation in vascular endothelial cells, but it remains unclear whether curcumin can alleviate the effect of inflammatory reactions in macrophage-derived foam cells while treated with degraded product of PLLA. In this study, PLLA-BVS was implanted in the porcine coronary artery to examine increased macrophages and inflammatory cytokines such as NF-κb and TNF-α by histology and immunohistochemistry. In vitro, macrophage-derived foam cells were induced by Ox-LDL and observed by Oil Red Staining. Foam cells were treated with pre-degraded PLLA powder, curcumin and PPARγ inhibitor GW9662, and the expression of IL-6, IL-10, TNF-α, NF-κb, PLA2 and PPARγ were investigated by ELISA or RT-qPCR. This study demonstrated that the macrophages and inflammatory factors increased after PLLA-BVS implantation in vivo, and foam cells derived from macrophages promoted inflammation by products of PLLA degradation in vitro. This present study was found that the inflammation of foam cells at the microenvironment of PLLA degraded products were significantly increased, and curcumin can attenuate the inflammation caused by the PLLA degradation via PPARγ signal pathway. In addition, curcumin should be further studied experimentally in vivo experiments on animal models as a potential therapeutic to reduce ISNA of PLLA-BVS.

**Figure:**
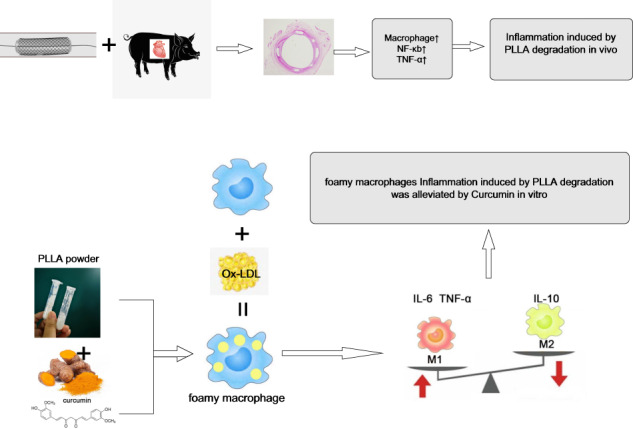
Graphical abstract

Graphical abstract

## Introduction

Coronary heart disease (CHD) still remains as a major contributor of death and disability globally [1]. Managing patients with coronary artery stenosis has substantially improved since percutaneous coronary intervention (PCI) [2]. Poly-L-lactic Acid (PLLA) as the most common use biodegradable materials has great application potential in PCI [3]. However, with the accumulation of clinical evidence, the problems of BVS such as thrombosis (ST) and in-stent restenosis (ISR) have been raised [4, 5]. In-stent neo-atherosclerosis (ISNA) has been identified as a major contributing factor to late stent failure resulting from ST and ISR [6–10]. ISNA is histologically characterized by an accumulation of lipid-laden foamy macrophages. The infiltration of foamy macrophages within the neointima results in the thinning of the fibrous cap to form thin-cap fibroatheroma, which may lead to ST and ISR [11]. Current research on ISNA is mostly derived from metallic scaffolds, and little is known about the progression of macrophage involvement in ISNA by PLLA scaffolds. A follow-up of 20 patients implanted with BVS by Moriyama et al. found that ISNA with luminal stenosis was observed 5 years after BVS implantation [12]. High Mw PLLA scaffold degraded completely in 2–3 years [13]. In fact, the residual struts in the pig coronary arteries were still found in our previous 4-year follow-up thought most of the struts were absorbed into the vascular walls [14]. For orthopedic patients, the degradation time may be longer [15]. In vivo, PLLA and its copolymers release low molecular weight polymers undergo hydrolysis and biodegradation, making a complicated and dynamic microenvironment [16, 17]. In our previous studies, pre-degraded low molecular PLLA powder was used to simulate the degradation products of PLLA. However, we have not studied the relationship between inflammation caused by macrophages and ISNA in the microenvironment of late degradation products. Macrophage is associated with foam cells formation and atherosclerotic instability by lipid accumulation and inflammatory cytokines. Therefore, targeting macrophage inflammation is a potential therapeutic strategy for atherosclerosis [18, 19]. As part of the traditional Chinese medicine, Berberine alleviates ox-LDL-induced macrophage activation by downregulating galectin-3 via the NF-κB and AMPK signaling pathways in ISNA after PCI [20]. Curcumin (Cur), an active pharmaceutical monomer extracted from ginger, has various pharmacological uses such as lipid lowering and anti-inflammation, and is widely implicated in the regulation of various signaling pathways such as fat transport and inflammation [21]. Accumulating evidences suggests that curcumin exert anti-atherosclerotic effect by decreasing the inflammatory responses of macrophage via NF-κb pathway as a PPARγ activator [22]. The effects of lipid-lowering and anti-inflammation of curcumin have been pointed out in reports, in which curcumin showed the benefits against hyperlipidemia and the development of atherosclerosis [23]. And curcumin inhibited the neointimal formation and reduced inflammatory responses in the animal model of carotid artery restenosis via Raf/MEK/ERK pathway [24]. However, the microenvironment of degraded polymers was not concerned enough. Some reports noted the encapsulation of curcumin in polymeric nanoparticles for anti-inflammatory therapy [25], but curcumin-mediated effects on the biocompatibility of polymers doesn’t cause enough concern. Some studies demonstrated that curcumin can reduced the inflammatory responses to bioresorbable PLLA fibers and may benefit PLLA-base implants [26]. And our previous study found that curcumin exerts anti-inflammatory effects during the PLLA degradation progress, related to the NF-κb signaling pathway, suggesting that curcumin has the potential to improve PLLA biocompatibility [17]. Here, we focused on the inflammatory responses and macrophages or foamy macrophages in the setting of PLLA in vivo and in vitro, further exploring the anti-inflammatory effect of curcumin via peroxisome proliferator-activated receptor gamma (PPARγ) signaling pathway.

## Materials and methods

### Materials

The following materials were used in this study: PLLA-BVS crimped on 3.0 × 15 mm balloon catheters (VasoTech, Inc. Lowell, MA, USA), Tibetan miniature pigs purchased from Pearl River Laboratory (Dongguan, China), Anti-NF-kB p65 antibody (Cell signaling, #6956, USA.) and Anti-TNF alpha antibody for IHC (Abcom, #ab6671, UK.), (Poly(L-lactide) OH (Mw 15,000–30,000 Da, Daigang Biomaterial Company, #DG-LOH050, China), human acute monocytic leukemia cell line, THP-1 (ZhongqiaoXinzhou, China #ZQ0086), RPMI 1640 (Gibco, #11875085), FBS (Sciencell, #0500, USA), penicillin-streptomycin (Gibco, #15140122, USA), phorbol-12-myristae-13-acetate (PMA, MCE, Shanghai, China, #HY-1879), Oxidized LDL (Ox-LDL, Shanghai Yuanye Bio-Technology, #S24879-2mg, China), Oil Red O solution (Sigma,#SLBW7964, USA.), Cell Counting Kit-8 (CCK-8, Dojindo, Kumamoto, Japan).curcumin (MCE, Shanghai, China, #HY-N0005), GW9662 (Abcam, #ab141261, UK.)ELISA kit for IL-6, TNF-α, IL-10 and Lp-PL-A2 (Meimian Company, China), Trizol RNA extraction kit (Shanghai Shenggong Institute of Biological Engineering, China, #A019-2), PrimeScript™ RT kit with GDNA-Eraser reverse transcription Kit (TAKARA, #RR047A), SYBR qPCR Mix Kit (Guangzhou Sihe, China, #SH3005).

### Methods

#### Animal preparation and scaffold implantation

Animal protocols approved by the Institutional Animal Care Committee at the Dongguan Affiliated Hospital of Jinan University, School of Medicine, and conformed with the “Guide for the Care and Use of Laboratory Animals” published by the US National Institutes of Health (NIH Publication number 85-23, revised 1996). PLLA-BVS crimped on 3.0 × 15 mm balloon catheters were sterilized with gamma radiation prior to implantation. 28 Tibetan miniature pigs, weighing 20–25 Kg, were implanted with PLLA stent in the coronary artery according to previously described procedures [27]. 28 Tibetan miniature pigs were divided into 4 groups according to the different follow-up time after implantation of the PLLA-BVS (10 pigs at 14th day, 10 pigs at 28th day, and 8 pigs at 90th day). Left anterior descending arteries with devices were carefully acquired in animals after excessive anesthesia. Left anterior descending artery segments from 3 healthy Tibetan miniature pigs were used as controls (0 day, before implantation).

#### Histological analysis and immunohistochemistry

All coronary arteries with implanted scaffold were paraffin-embedded and serial cross-sections were obtained at 4 μm thick for histological staining. The sections were then stained with hematoxylin eosin (HE) and NF-κb, TNF-α were detected by immunohistochemistry as described previously [27].

#### Preparation of pre-degraded PLLA powder

High Mw PLLA (>600,000 Da) is used clinically for the fabrication of vascular stents, which are completely degraded in 2 or 3 years. For in vitro assays, low Mw PLLA powder is commonly to mimic the later products in a short time in vitro in experiments. PLLA with Mw 15,000 Da predegraded for 16 weeks was chosen because of high lactic acid content during degradation in our previous studies [17]. In brief, after sterilization by UV irradiation, the Mw 15,000 PLLA powder was weighted accurately with an electronic balance (Sartorius, BSA223S, Beijing, China) and dissolved in sterile saline at the suggested concentration of 0.2 g/ml, based on ISO 10993-12. The degradation products were collected after incubation in a shaking incubator (THZ-100, Yiheng, China) at 37 °C and 100 rpm for 16 weeks. After centrifugation (Eppendorf, #5804 R; 5000 rpm for 5 min), drying and grinding, the remaining degradation products were collected.

#### Cell culture

THP-1 cells were cultured in the medium (RPMI 1640 supplemented with 10% FBS and 1% Penicillin-streptomycin), at 37 °C, 5% CO_2_. They were passaged every 48 h. Macrophages were then induced from 20 × 10^4^/ml THP-1 cells in media containing PMA at 100 ng/ml. The PMA-containing media was then replaced with several kinds of basic medium containing 25, 50, 100, 150 ug/ml Ox-LDL respectively, and cells cultured for 48 h to establish the THP-1 macrophage-derived foam cells. Foam cell formation was examined using oil red O staining as described before [28]. Briefly, foam cells derived by culturing macrophages for 48 h in ox-LDL were fixed with 4% PFA at 25 °C overnight and washed with 0.01 M PBS. Cell morphology was then evaluated under the micrograph after staining with oil red O at 37 °C for 30 min. As long as lipid droplets can be clearly observed under the microscope, the foam cells induced by the lowest concentration of Ox-LDL should be selected for subsequent experiments to avoid too strong inflammatory response. Macrophage-derived foam cells were divided into 6 groups, Blank control group (Con group), PLLA group (foam cells treated with pre-degraded PLLA), PLLA + Cur group (foam cells treated with PLLA and curcumin) and PLLA + Cur+GW9662 group (foam cells treated with PLLA, curcumin and GW9662), Cur group (foam cells treated with curcumin alone), Cur+GW9662 group (foam cells treated with curcumin and GW9662). PLLA powder was in 1640 medium after sterilization by UV irradiation. 100 mg curcumin dissolved in 2.715 mL DMSO was dissolved to obtain a concentration of 100 mmol/L in accordance with the instructions, then diluted to 1 mmol/L concentration of curcumin with the cell medium, and packed separately and stored at −80 °C. After the cytotoxicity test, 10 mg/mL PLLA and 20 μmol/L curcumin were used in subsequent experiments. GW9662 was used in this study after cytotoxicity assessment with CCK8 according to the instructions. Foam cells were cultured with degraded products of PLLA, curcumin and GW9662 for downstream experiments.

#### ELISA for IL-6, TNF-α, IL-10 and LP-PLA2 detection

To assess the effects of curcumin on inflammation in foam cells, the release levels of IL-6, TNF-α, IL-10, and Lp-PLA2 in supernatants of foam cells cultured in product of PLLA degradation for 24 h were measured by ELISA following kit manufacturer instructions.

#### RT-qPCR analysis of IL-6, TNF-α, IL-10, PPARγ

RNA extraction, reverse transcription and RT-qPCR were done according to kit instructions. The following primers were designed using Premier 5 and synthesized by Guangzhou Jierui Biological Company (China):

IL-6: F: atgaactccttctccacaagcgc, R: gaagagccctcaggctggactgg,

TNF-α: F: cagcctcttctccttcctga, R: ggaagacccctcccagataga,

IL-10: F - ggttgtcgtctcattctgaaaga, R: ggtagaggacccaagttcgttaaga,

PPARγ: F: agcaacagtcatccataaaag, R: acatccccacagcaaggcatt.

GAPDH: F: accaccatggagaaggctgc, R: ctcagtgtagcccaggatgc.

#### Statistical analysis

Data are expressed as mean ± SD. GraphPad prism 6.0 and SPSS 17.0 were used for statistical analyses. One-way ANOVA was used for multiple comparisons. *P* = < 0.05 indicates statistically significant differences.

## Results

### Histology and immunohistochemistry of porcine coronary arteries implanted with PLLA stents follow-up to 90d

**Fig. 1:**
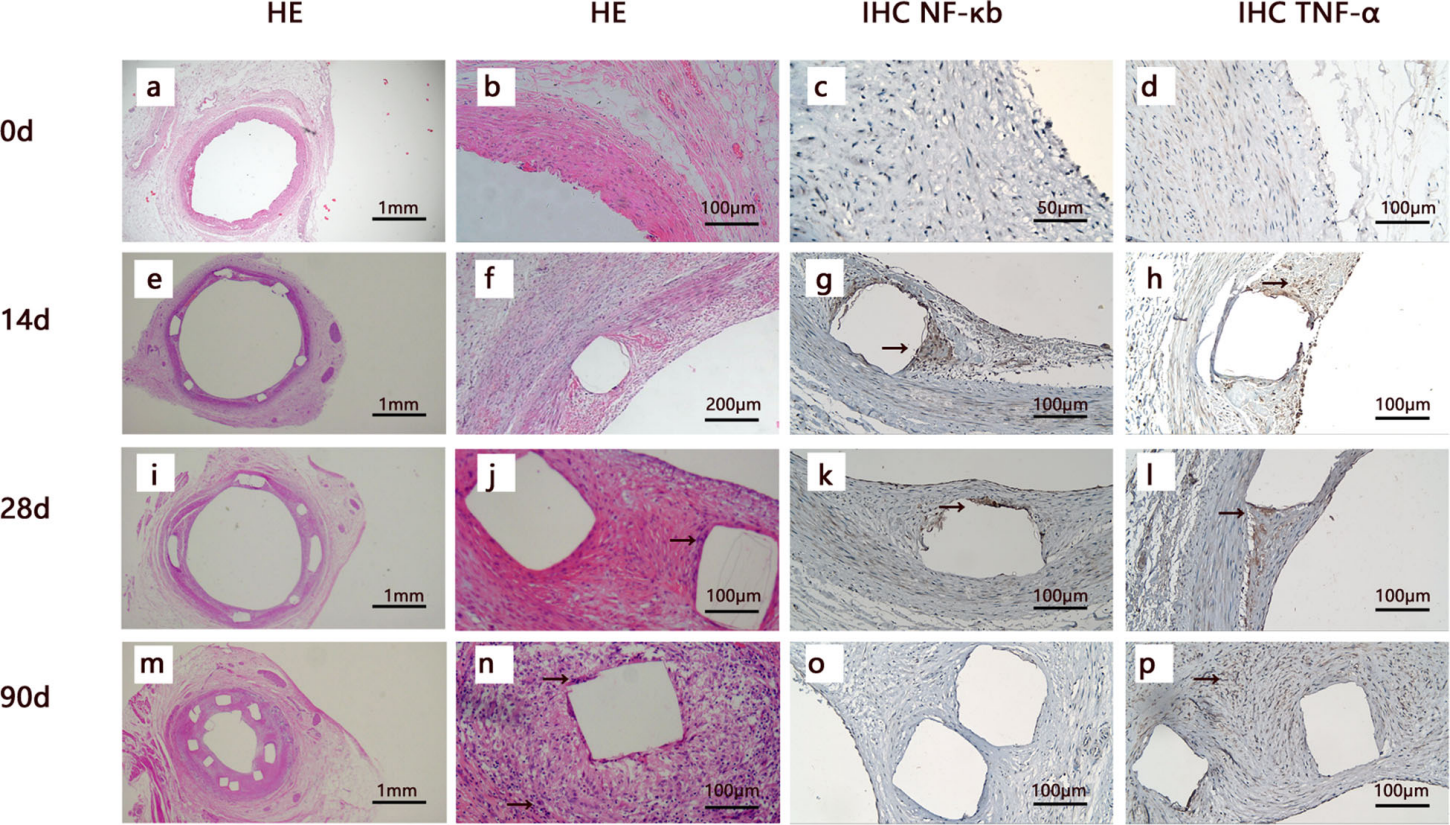
Histology and immunohistochemistry of porcine coronary arteries implanted with PLLA-BVS follow-up to 90 d. The images were shown by HE staining (from left to right, first and second columns) and immunohistochemistry staining (third and fourth columns) of the coronary arteries after implantation for 0 d (**a–d**), 14 d (**e–h**), 28 d (**i–l**), 90 d (**m–p**), respectively. White squares in all pictures indicate components of PLLA stent, macrophages were indicated by black arrows at 28 and 90th day (**j**, **n**) in HE stain image, and positive immunohistochemistry staining of NF-κb/TNF-α were indicated by black arrows at 14 d, 28 d and 90 d (**g**, **h**, **k**, **l**, **p**).

Macrophage infiltration could be observed around the scaffolds after PLLA-BVS implanted for 90 days. Immunohistochemistry showed that the expression of NF-κb and TNF-α increased in coronary artery implanted with PLLA-BVS indicating inflammation in the endothelium and surrounding the PLLA stent after implantation for 14, 28 and 90 days. (Fig. 1)

The images were shown by HE staining (from left to right, first and second columns) and immunohistochemistry staining (third and fourth columns) of the coronary arteries after implantation for 0 d (a–d), 14 d (e–h), 28 d (i–l), 90 d (m–p), respectively. White squares in all pictures indicate components of PLLA stent, macrophages were indicated by black arrows at 28 and 90th day (j & n) in HE stain image, and positive immunohistochemistry staining of NF-κb/TNF-α were indicated by black arrows at 14 d, 28 d and 90 d (g, h, k, l and p).

### Formation of foam cells derived from THP-1 macrophages and Oil Red O staining

**Fig. 2:**
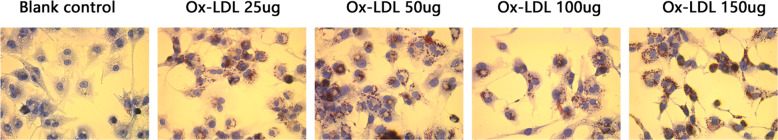
Results of the positive staining of Oil Red O in foam cells treated with 25, 50, 100, 150 ug/ml Ox-LDL. Compared with blank control, the lipid drops in foam cells co-incubated with 25 ug/ml Ox-LDL were clear enough

As shown in Fig. 2, the lipid drops in foam cells induced with different concentration of Ox-LDL were noted brown positive staining after Oil Red O staining, indicating successful foam cell formation, and foam cells induced by 25 ug/ml Ox-LDL were applied for the next experiment.

### Effect of GW9662 on cell viability in macrophages

**Fig. 3:**
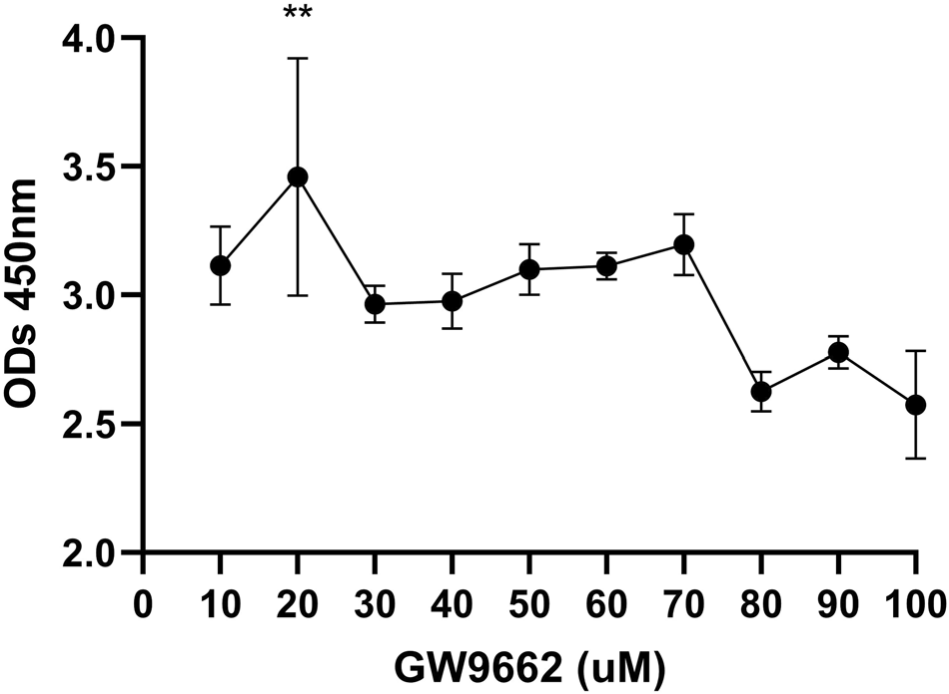
ODs of macrophages in different concentration of GW9662. Note ODs of 20 μm GW9662 was highest in all the groups

To assess the effects on cell viability of GW9662 in macrophages, CCK-8 was used (Fig. 3). Compared with other groups, ODs of macrophages in 20 uM GW9662 was higher at 24 h.

### Curcumin attenuates foam cells Inflammation associated with Poly-L-lactic acid degradation

**Fig. 4:**
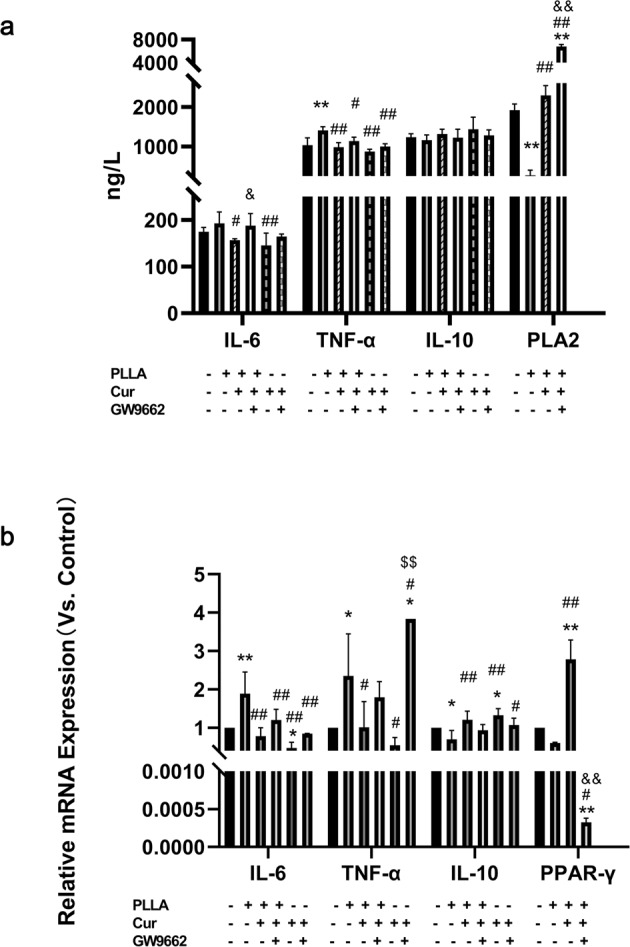
Measurement results of inflammatory cytokines and PPARγ pathway signal by ELISA and RT-qPCR. **a** The release level of IL-6, TNF-α, IL-10 and LP-PLA2 detected by ELISA. The level of IL-6 and TNF-α in PLLA group significantly increased while comparing to the Con group, and decreased while comparing to the PLLA + Cur group. Compared with the PLLA + Con group, the levels of Lp-PLA2 were decreased in PLLA group. Compared with PLLA group, Lp-PLA2 increased in PLLA + Cur group. And compared with PLLA + Cur group, Lp-PLA2 increased in PLLA + Cur+GW9662 group. There were no significantly differences among each group of release levels of IL-10. **b** The mRNA level of IL-6, TNF-α, IL-10 and PPARγ detected by RT-qPCR. Compare to Con group, the levels of IL-6 and TNF-α mRNA significantly increased in PLLA group and decreased in PLLA + Cur group; the level of IL-10 and PPAR-γ mRNA significantly decreased in PLLA group and increased in PLLA + Cur group. In contrast, the level of PPAR-γ mRNA decreased in PLLA + Cur+GW9662 group while compare to PLLA + Cur group. (Compared with Con group, **p < 0.01, *p = < 0.05, respectively. Compared with PLLA group, ##*p* < 0.01, #*p* = < 0.05, respectively. Compared with PLLA + Cur group, &&*p* < 0.01, &*p* = < 0.05, respectively. Compared with Cur group, $$*p* < 0.01, $*p* = < 0.05, respectively)

After cultured for 24 h, we analyzed the release level in supernatant and mRNA expression level of cells of inflammatory factors like IL-6, TNF-α and IL-10 in each group using ELISA and RT-qPCR, respectively. As an inflammatory factor closely related to the inflammatory activity of atherosclerotic plaques, Lp-PLA2 in the supernatant of cultured cells was also detected by ELISA. As is show to Fig. 4a, the secretion of TNF-α in the PLLA group was significantly higher than Con group. There is no significant difference in the secretion level of the anti-inflammatory cytokine, IL-10 in each group. RT-qPCR analysis revealed that compared to Con group, IL-6 and TNF-α mRNA level was significantly elevated, while IL-10 mRNA level was significantly reduced in the PLLA group (Fig. 4b). Meanwhile, the IL-6 and TNF-α mRNA level was significantly reduced, while IL-10 mRNA level was significantly elevated in the PLLA + Cur group. After the addition of GW9662, the levels of pro-inflammatory cytokines such as IL-6 in PLLA + Cur group had a downward trend (Fig. 4a), while the anti-inflammatory cytokine IL-10 was in the fall both in ELISA and RT-qPCR results. However, there were no significant difference in the two groups. IL-6 and TNF-α decreased and IL-10 increased significantly in Cur group while comparing with blank control group. And IL-6 increased significantly in Cur + GW9662 group while comparing with Cur group. Additionally, TNF-α increased significantly in Cur+PLLA group while comparing with Cur group. Interesting, the levels of Lp-PLA2 were decreased in PLLA group and increased in PLLA + Cur group and PLLA + Cur + GW9662 group.

### PPARγ signaling participates in curcumin-induced anti-inflammation in foam cells associated with Poly-L-lactic acid degradation

To investigate the effect of PPARγ signaling pathway on curcumin in the inflammatory reactions of macrophage-derived foam cells, we evaluated the mRNA level of PPARγ in 4 groups (PLLA group, PLLA + Cur group, PLLA + Cur + GW9662 group). PPARγ expression levels significantly increased in Cur group and reduced in GW9662 group (Fig. 4b).

## Discussion

Among the available biodegradable biomaterials, PLLA is considered to be a promising candidate for biodegradable scaffolds due to its excellent chemical and mechanical properties which have great application potential in PCI. Whereas the increasing target lesion failure and device thrombosis with the PLLA-BVS in clinical trials hinder its application. The published data showed that the complete degradation time in vivo exceeded expectations, and In-stent Neoatherosclerosis (ISNA) with luminal stenosis was observed 5 years after PLLA-BVS implantation [12]. The accumulation of PLLA degradation products and inflammatory reactions were important contributing factors for this result [29, 30]. Macrophages play an important role in the PLLA degradation process by phagocytosing the polymer microparticles. In our previous reports, the inflammatory responses in artery endothelial cells [17] treated with the low molecular weight PLLA degradation extract or solid product were increased and reversed after the addition of curcumin. In the present study, macrophages and inflammatory cytokines surrounding PLLA scaffold strut in animal in vivo were observed, and the release and expression level of inflammatory cytokines in macrophage-derived foam cells treated with pre-degraded low molecular weight PLLA products were studied iv vitro. As an anti-inflammatory medicine and a regulator of macrophage polarization, curcumin was then implied to macrophage to reveal the molecular mechanisms such as PPARγ pathway.

The expression of NF-κb/TNF-α increased and macrophages surrounding PLLA were found after PLLA stents were implanted in the artery in this study, suggesting inflammatory response caused by PLLA should be monitored during the acceleration of degradation. In-stent neoatherosclerosis (ISNA) is considered to be one of the major factors in the late failure of biodegradable scaffolds implant [7–10]. Macrophages play an important role at all stages of atherosclerotic lesion development [31]. A crucial step in atherosclerosis progression is inflammatory response by foam cells in the lipid core and foam cells in macrophage-derived plaques exhibit similar polarization by secreting pro-inflammatory cytokines [23]. The main cause of foam cells generation is the excessive influx of modified low-density lipoproteins (LDL) and accumulation of cholesterol esters in intimal macrophages [32]. Numerous factors are involved in the development of plaque [19]. Cytokines like IL-6, TNF-α and IL-10 promote the development of neoatherosclerosis [33]. Lipoprotein-associated phospholipase A2 (Lp-PLA2), a vascular specific biomarker, plays a role in the development of atherosclerotic lesions and formation of a necrotic core, leading to more vulnerable plaques [34].

In our study, foam cell dominated inflammatory response was promoted by PLLA. Macrophages play an important role at all stages of atherosclerotic lesion development. They have different roles in proinflammatory M1 macrophages (releasing IL-6 and TNF-α) and anti-inflammatory M2 macrophages (releasing IL-10). In general, the M1 contributes to onset of inflammation, whereas the M2 orchestrates resolution and repair [31]. Interestingly, the level of special vascular inflammatory factor Lp-PLA2 in the PLLA group has not increased. We speculate that there also have a significant number of macrophages engulf PLLA particles when macrophages uptake ox-LDL. These macrophages were absence of expression of factors and markers of foam cells, and it need to further studies in the future. In this study, we didn’t detect ISNA in coronary after implant PLLA-BVS follow-up to 90 days. This may be related to the animals we chose were healthy pigs and high fat diet feeding was not performed in this study. Furthermore, the follow-up time is still too short for ISNA formation [12, 35].

Curcumin has been extensively studied as a potential anti-atherosclerosis agent. Mounting evidence indicates that curcumin has anti-inflammation effects and can inhibit lipid accumulation. However, its effect on inflammatory reactions in macrophage-derived foam cells associated with the product of pre-degraded PLLA is unclear. IL-6 has been reported as a biomarker for plaque initiation and destabilization [36]. IL-10 deficiency is reported to increase atherosclerotic plaque size and promote atherosclerosis development. Human IL-10 gene transfer decreased atherosclerosis and atherosclerotic plaque instability [37]. Here, we made similar observations. The level of IL-6 and TNF-α were significantly decreased in the PLLA + Cur group, suggesting that curcumin may inhibit plaque development. On the contrary, curcumin stabilized the plaque by enhanced IL-10 expression by foam cells.

To determine if the effect of curcumin on inflammatory reactions is related to PPARγ pathway, we measured its mRNA levels. Mounting evidence suggests curcumin activates PPARγ during macrophage polarization and that NF-κb plays a key role in the PPARγ pathway in anti-inflammatory reactions [38, 39]. Synthetic antagonists of the nuclear receptor PPARγ such as GW9662 are widely used to elucidate receptor-mediated ligand effects. Here, we found increased PPARγ expression in curcumin-treated foam cells (PLLA + Cur group). On the contrary, suppressed PPARγ expression was founded in GW9662 group indicating that curcumin may modulate inflammatory reactions in lipid forming cells via PPARγ signaling.

At the same time, the inflammatory responses significantly decreased in Cur group and Cur + PLLA group demonstrated the anti-inflammatory effect of the Cur in foam cells treated with PLLA. Furthermore, the anti-inflammatory effect of Cur reversed by the PPARγ blocker-GW9662, as was show in the PLLA + Cur + GW9662 and Cur + GW9662 group, which indicated that Cur attenuates the inflammation of foam cells via PPARγ signal pathway. Those results were trying to show the conclusion that curcumin can attenuate the inflammation caused by the PLLA degradation via PPARγ signal pathway.

## Conclusions

This study demonstrated that the inflammatory factors release and macrophages surrounding PLLA scaffold were obvious in vivo, and the inflammatory responses in macrophages-derived foam cells promoted by PLLA pre-degradation product in vitro. Curcumin represents a promising agent to reduce ISNA of PLLA-BVS by alleviating foam cell inflammation caused by PLLA degradation via PPARγ pathway activation. In addition, inflammation was also observed in vivo, but more details are needed to study for exploring the complex reasons.

## Limitation

A number of limitations need to be noted regarding this study. Firstly, some cytokines, like Lp-PLA2 and PPARγ were not measured in all groups in vitro experiment adequately, and more in-depth studies are needed to validate thethe molecular mechanisms of curcumin’s effects. Secondly, curcumin was not used in the in vivo experiments in this study, which should be considered as a supplementary part for future research.

## References

[1] WHO. Cardiovascular diseases (CVDs). 2021. https://www.who.int/en/news-room/fact-sheets/detail/cardiovascular-diseases-(cvds). Accessed 11 June 2021.

[2] Gaba P, Gersh BJ, Ali ZA, Moses JW, Stone GW. Complete versus incomplete coronary revascularization: definitions, assessment and outcomes. NAT REV CARDIOL. 2021; 10.1038/s41569-020-00457-5 10.1038/s41569-020-00457-5 33067581

[3] Verdoia M, Kedhi E, Suryapranata H, Galasso G, Dudek D, De Luca G. Poly (l-lactic acid) bioresorbable scaffolds versus metallic drug-eluting stents for the treatment of coronary artery disease: A meta-analysis of 11 randomized trials. Catheter Cardiovasc Interv. 2020; 10.1002/ccd.28594 10.1002/ccd.28594 31730255

[4] Peng X, Qu W, Jia Y, Wang Y, Yu B, Tian J. Bioresorbable Scaffolds: Contemporary Status and Future Directions. Front Cardiovasc Med. 2020; 10.3389/fcvm.2020.589571 10.3389/fcvm.2020.589571 PMC7733966 33330651

[5] Wykrzykowska JJ, Kraak RP, Hofma SH, van der Schaaf RJ, Arkenbout EK, IJsselmuiden AJ, et al. Bioresorbable Scaffolds versus Metallic Stents in Routine PCI. N Engl J Med. 2017; 10.1056/NEJMoa1614954 10.1056/NEJMoa1614954 28402237

[6] Borovac JA, D’Amario D, Vergallo R, Porto I, Bisignani A, Galli M, et al. Neoatherosclerosis after drug-eluting stent implantation: a novel clinical and therapeutic challenge. Eur Heart J Cardiovasc Pharmacother. 2019; 10.1093/ehjcvp/pvy036 10.1093/ehjcvp/pvy036 30285099

[7] Moriyama N, Shishido K, Tanaka Y, Laine M, Saito S. Neoatherosclerosis- Long-Term Assessment of Bioresorbable Vascular Scaffold. Circ Rep. 2019; 10.1253/circrep.CR-19-0100 10.1253/circrep.CR-19-0100 PMC7897685 33693100

[8] Hiltrop N, Desmet W, Adriaenssens T, Bennett J. Neoatherosclerosis: an emerging and conceptually unexpected cause of very late bioresorbable vascular scaffold failure. Eurointervention. 2017; 10.4244/EIJ-D-16-00259 10.4244/EIJ-D-16-00259 28317791

[9] Kang SH, Kang SJ, Kim WJ. Neoatherosclerosis as the Cause of Late Failure of a Bioresorbable Vascular Scaffold at 8 Months. JACC Cardiovasc Interv. 2017; 10.1016/j.jcin.2017.07.018

[10] Mangiameli A, Ohno Y, Attizzani GF, Capodanno D, Tamburino C. Neoatherosclerosis as the cause of late failure of a bioresorbable vascular scaffold. JACC Cardiovasc Interv. 2015; 10.1016/j.jcin.2014.11.014 10.1016/j.jcin.2014.11.014 25819179

[11] Otsuka F, Byrne RA, Yahagi K, Mori H, Ladich E, Fowler DR, et al. Neoatherosclerosis: overview of histopathologic findings and implications for intravascular imaging assessment. Eur Heart J. 2015; 10.1093/eurheartj/ehv205 10.1093/eurheartj/ehv205 25994755

[12] Moriyama N, Shishido K, Tanaka Y, Yokota S, Hayashi T, Miyashita H, et al. Neoatherosclerosis 5 years after bioresorbable vascular scaffold implantation. J Am Coll Cardiol. 2018; 10.1016/j.jacc.2018.02.051 10.1016/j.jacc.2018.02.051 29699614

[13] Onuma Y, Serruys PW, Perkins LE, Okamura T, Gonzalo N, Garcia-Garcia HM, et al. Intracoronary optical coherence tomography and histology at 1 month and 2, 3, and 4 years after implantation of everolimus-eluting bioresorbable vascular scaffolds in a porcine coronary artery model: an attempt to decipher the human optical coherence tomography images in the ABSORB trial. Circulation. 2010; 10.1161/CIRCULATIONAHA.109.921528 10.1161/CIRCULATIONAHA.109.921528 20975003

[14] Chen D, Dong Z, Xi Y, Chen C, Zhang S, Zeng S, et al. Long-Term Arterial remodeling after bioresorbable scaffold implantation 4-year follow-up of quantitative coronary Angiography, Histology and Optical Coherence Tomography. Cardiovasc Eng Technol. 2020; 10.1007/s13239020-00495-7 10.1007/s13239-020-00495-7 33108646

[15] Amini AR, Wallace JS, Nukavarapu SP. Short-term and long-term effects of orthopedic biodegradable implants. J Long Term Eff Med Implants. 2011; 10.1615/jlongtermeffmedimplants.v21.i2.10 10.1615/jlongtermeffmedimplants.v21.i2.10 PMC3470866 22043969

[16] Wang QS, Cui YL, Gao LN, Guo Y, Li RX, Zhang XZ. Reduction of the pro-inflammatory response by tetrandrine-loading poly(L-lactic acid) films in vitro and in vivo. J Biomed Mater Res A. 2014; 10.1002/jbm.a.35083 10.1002/jbm.a.35083 24442958

[17] Chen D, Weng L, Chen C, Zheng J, Wu T, Zeng S, et al. Inflammation and dysfunction in human aortic endothelial cells associated with poly-l-lactic acid degradation in vitro are alleviated by curcumin. J Biomed Mater Res A. 2019; 10.1002/jbm.a.36778 10.1002/jbm.a.36778 31408261

[18] Wang D, Yang Y, Lei Y, Tzvetkov NT, Liu X, Yeung A, et al. Targeting Foam Cell Formation in Atherosclerosis: Therapeutic Potential of Natural Products. Pharmacol Rev. 2019; 10.1124/pr.118.017178 10.1124/pr.118.017178 31554644

[19] Liu X, Wu J, Tian R, Su S, Deng S, Meng X. Targeting foam cell formation and macrophage polarization in atherosclerosis: The Therapeutic potential of rhubarb. Biomed Pharmacother. 2020; 10.1016/j.biopha.2020.110433 10.1016/j.biopha.2020.110433 32768936

[20] Pei C, Zhang Y, Wang P, Zhang B, Fang L, Liu B, et al. Berberine alleviates oxidized low-density lipoprotein-induced macrophage activation by downregulating galectin-3 via the NF-kappaB and AMPK signaling pathways. Phytother Res. 2019; 10.1002/ptr.6217 10.1002/ptr.6217 PMC6587449 30402951

[21] Cao J, Ye B, Lin L, Tian L, Yang H, Wang C, et al. Curcumin Alleviates oxLDL Induced MMP-9 and EMMPRIN Expression through the Inhibition of NF-kappaB and MAPK Pathways in Macrophages. Front Pharmacol. 2017; 10.3389/fphar.2017.00062 10.3389/fphar.2017.00062 PMC5306337 28261097

[22] Mohammadian HS, Karimzadeh MR, Azhdari S, Vahedi P, Abdollahi E, Momtazi-Borojeni AA. Modulatory effects of curcumin on the atherogenic activities of inflammatory monocytes: Evidence from in vitro and animal models of human atherosclerosis. Biofactors. 2020; 10.1002/biof.1603 10.1002/biof.1603 31875344

[23] Momtazi-Borojeni AA, Abdollahi E, Nikfar B, Chaichian S, Ekhlasi-Hundrieser M. Curcumin as a potential modulator of M1 and M2 macrophages: new insights in atherosclerosis therapy. Heart Fail Rev. 2019; 10.1007/s10741-018-09764-z 10.1007/s10741-018-09764-z 30673930

[24] Zhang D, Yang Y, Li Y, Zhang G, Cheng Z. Inhibitory Effect of Curcumin on Artery Restenosis Following Carotid Endarterectomy and Its Associated Mechanism in vitro and in vivo. Drug Des Devel Ther. 2020; 10.2147/DDDT.S229607 10.2147/DDDT.S229607 PMC7049773 32161445

[25] Mahmood K, Zia KM, Zuber M, Salman M, Anjum MN. Recent developments in curcumin and curcumin based polymeric materials for biomedical applications: A review. Int J Biol Macromol. 2015; 10.1016/j.ijbiomac.2015.09.026 10.1016/j.ijbiomac.2015.09.026 26391597

[26] Su SH, Nguyen KT, Satasiya P, Greilich PE, Tang L, Eberhart RC. Curcumin impregnation improves the mechanical properties and reduces the inflammatory response associated with poly(L-lactic acid) fiber. J Biomater Sci Polym Ed. 2005; 10.1163/1568562053654077 10.1163/1568562053654077 15850289

[27] Lan Z, Lyu Y, Xiao J, Zheng X, He S, Feng G, et al. Novel biodegradable drug-eluting stent composed of poly-L-lactic acid and amorphous calcium phosphate nanoparticles demonstrates improved structural and functional performance for coronary artery disease. J Biomed Nanotechnol. 2014; 10.1166/jbn.2014.1868 10.1166/jbn.2014.1868 24804540

[28] Li X, Guo D, Chen Y, Hu Y, Zhang F. Effects of Altered Levels of Pro- and Anti-Inflammatory Mediators on Locations of In-Stent Reocclusions in Elderly Patients. Mediators Inflamm. 2020; 10.1155/2020/1719279 10.1155/2020/1719279 PMC7530477 33029103

[29] Wang X, Zachman AL, Chun YW, Shen FW, Hwang YS, Sung HJ. Polymeric stent materials dysregulate macrophage and endothelial cell functions: implications for coronary artery stent. Int J Cardiol. 2014; 10.1016/j.ijcard.2014.04.228 10.1016/j.ijcard.2014.04.228 PMC4070878 24820736

[30] Bergsma EJ, Rozema FR, Bos RR, de Bruijn WC. Foreign body reactions to resorbable poly(L-lactide) bone plates and screws used for the fixation of unstable zygomatic fractures. J Oral Maxillofac Surg. 1993; 10.1016/s0278-2391(10)80267-8 10.1016/s0278-2391(10)80267-8 8492205

[31] Mazidi M, Karimi E, Meydani M, Ghayour-Mobarhan M, Ferns GA. Potential effects of curcumin on peroxisome proliferator-activated receptor-gamma in vitro and in vivo. World J Methodol. 2016; 10.5662/wjm.v6.i1.112 10.5662/wjm.v6.i1.112 PMC4804246 27019802

[32] Hutchins PM, Heinecke JW. Cholesterol efflux capacity, macrophage reverse cholesterol transport and cardioprotective HDL. Curr Opin Lipidol. 2015; 10.1097/MOL.0000000000000209 10.1097/MOL.0000000000000209 PMC4617325 26270810

[33] Chistiakov DA, Melnichenko AA, Myasoedova VA, Grechko AV, Orekhov AN. Mechanisms of foam cell formation in atherosclerosis. J Mol Med (Berl). 2017; 10.1007/s00109-017-1575-8 10.1007/s00109-017-1575-8 28785870

[34] Tselepis AF, Rizzo M, Goudevenos IA. Therapeutic modulation of lipoprotein-associated phospholipase A2 (Lp-PLA2). Curr Pharm Des. 2011; 10.2174/138161211798220936 10.2174/138161211798220936 22074435

[35] Daugherty A, Tall AR, Daemen M, Falk E, Fisher EA, Garcia-Cardena G, et al. Recommendation on Design, Execution, and Reporting of Animal Atherosclerosis Studies: A Scientific Statement From the American Heart Association. Arterioscler Thromb Vasc Biol. 2017; 10.1161/ATV.0000000000000062 10.1161/ATV.0000000000000062 28729366

[36] Poznyak AV, Wu WK, Melnichenko AA, Wetzker R, Sukhorukov V, Markin AM, et al. Signaling Pathways and Key Genes Involved in Regulation of foam Cell Formation in Atherosclerosis. Cells-Basel. 2020; 10.3390/cells9030584 10.3390/cells9030584 PMC7140394 32121535

[37] Shakeri F, Boskabady MH. Anti-inflammatory, antioxidant, and immunomodulatory effects of curcumin in ovalbumin-sensitized rat. Biofactors. 2017; 10.1002/biof.1364 10.1002/biof.1364 28509396

[38] Kahkhaie KR, Mirhosseini A, Aliabadi A, Mohammadi A, Mousavi MJ, Haftcheshmeh SM, et al. Curcumin: a modulator of inflammatory signaling pathways in the immune system. Inflammopharmacology. 2019; 10.1007/s10787-019-00607-3 10.1007/s10787-019-00607-3 31140036

[39] Zhang HF, Wu MX, Lin YQ, Xie SL, Huang TC, Liu PM, et al. IL-33 promotes IL-10 production in macrophages: a role for IL-33 in macrophage foam cell formation. Exp Mol Med. 2017; 10.1038/emm.2017.183 10.1038/emm.2017.183 PMC5704190 29099095

